# Media Health Literacy, eHealth Literacy, and the Role of the Social Environment in Context

**DOI:** 10.3390/ijerph15081643

**Published:** 2018-08-03

**Authors:** Diane Levin-Zamir, Isabella Bertschi

**Affiliations:** 1 Department of Health Education and Promotion, Clalit Health Services, Tel Aviv 62098, Israel; 2 School of Public Health, University of Haifa, Haifa 31905, Israel; 3 Department of Psychology, University of Zurich, Zürich 8050, Switzerland; isabella.bertschi@psychologie.uzh.ch

**Keywords:** health literacy, Media Health Literacy, eHealth Literacy, social environment, health apps, social support, digital health, empowerment

## Abstract

Health literacy describes skills and competencies that enable people to gain access to, understand and apply health information to positively influence their own health and the health of those in their social environments. In an increasingly media saturated and digitized world, these skill sets are necessary for accessing and navigating sources of health information and tools, such as television, the Internet, and mobile apps. The concepts of Media Health Literacy (MHL) and eHealth Literacy (eHL) describe the specific competencies such tasks require. This article introduces the two concepts, and then reviews findings on the associations of MHL and eHL with several contextual variables in the social environment such as socio-demographics, social support, and system complexity, as a structural variable. As eHL and MHL are crucial for empowering people to actively engage in their own health, there is a growing body of literature reporting on the potential and the effectiveness of intervention initiatives to positively influence these competencies. From an ethical standpoint, equity is emphasized, stressing the importance of accessible media environments for all—including those at risk of exclusion from (digital) media sources. Alignment of micro and macro contextual spheres will ultimately facilitate both non-digital and digital media to effectively support and promote public health.

## 1. Introduction

Several factors have led, and continue to lead, to the development of health systems that enable, but also partly expect their users to adopt a much more active role in their health management than was customary some decades ago. The empowerment of groups and individuals to engage in their own health, for example by shared decision-making with health professionals, or by adoption of health-promoting lifestyles, is an important goal of public health in the 21st century and a priority in the UN Sustainable Development Goals. Being able to actively manage one’s health is very demanding of citizens. It is largely, although by no means entirely, dependent on the availability, accessibility, and appropriateness of health information. To reflect the skill set required to effectively manage health and navigate the health system from health care to disease prevention and health promotion, the concept of health literacy was developed. A wide variety of definitions exist, but in general Health literacy (HL) is conceptualized as skills and competences enabling people to obtain and interpret health information and apply their knowledge to inform health-related decision-making (for an overview of definitions see e.g., [1,2]).

In an increasingly media saturated and digital environment, a large proportion of health-related messages and information today is circulated and accessed through the media and digital sources. Thus, researchers together with health practitioners have developed two closely linked, but nonetheless distinct concepts related to HL: *Media Health Literacy* [3] and *eHealth Literacy* [4]. Media Health Literacy and eHealth Literacy have both proven to be associated with health information seeking and with health outcomes such as health behavior and health status across various population groups. Environmental factors linked to the social, organizational or economic context play an important role (a) in shaping individual, group or population MHL and eHL skills and (b) by posing specific demands on the situations in which such skills are required by the individual or group.

This article aims to introduce readers to the concepts of Media Health Literacy and eHealth Literacy, emphasizing their role in the social environment while demonstrating how context variables are relevant when applying the concepts in research and practice. We will critically discuss issues related to the two concepts and explore the ethical aspects of these concepts in research, practice, and policy.

## 2. The Concepts of Media Health Literacy and eHealth Literacy

Media Health Literacy (MHL) [3] is based on and builds on the synthesis of health literacy and media literacy [5]—two essential concepts for understanding the scope and significance of eHealth Literacy. The concept of Media Health Literacy is unique in that it takes into consideration not only information that has been communicated through the media to offer health guidance; but it also considers implicit and explicit mass media content commonly generated by commercial entities or health systems that can be either health-promoting or health-compromising. Based on the typology of the Nutbeam model of Health Literacy [6], Media Health Literacy is conceptualized as a continuum, ranging from (1) the ability to identify health-related content (explicit and/or implicit) in the various types of media; (2) recognize its influence on health behavior; (3) critically analyze the content (comparable to Critical Health Literacy), and (4) express intention to respond through action measured through personal health behavior or advocacy (comparable to Interactive Health Literacy). Thus, the validated measure of Media Health Literacy is comprised of these four categories and was shown to be highly correlated with health empowerment. As such, Media Health Literacy can be considered the precursor to eHealth literacy and is highly relevant for both non-digital (television, print, radio, etc.) and digital media (Internet, social media, and mobile tools).

While media in general has long since been recognized as the only social institution that accompanies the individual throughout the entire life course [7], over the past decade, digital media has received particular attention with regards to use for health purposes. The number of digital health offers has grown with impressive speed—an annual growth rate of about 25%. According to data from Research2Guidance, approximately 325,000 health apps were available in 2017, with 78,000 new mobile health applications being released between 2016 and 2017. Although it has been shown that only 7% of mHealth apps have more than 50,000 monthly active users, usage proportions are very likely to increase significantly in the near future [8]. The growing importance of digital media has led researchers, practitioners, and policy makers to reflect on the skills necessary for users, and the challenges they face to achieve effective outcomes, namely navigating the services, accessing relevant health information and adopting lifestyle changes. Well over a decade ago, Norman and Skinner [4] as pioneers in the field introduced the term eHealth Literacy (eHL) meaning ”the ability to seek, find, understand, and appraise health information from electronic sources and apply the knowledge gained to addressing or solving a health problem” (p. 1). They also developed a measurement tool for eHealth Literacy that has been used in many different settings around the globe, the eHealth Literacy Scale (eHEALS) [9]. It consists of eight items for which respondents self-rate their ease and skills when navigating Internet sources for valid health information. The original English scale has been translated into many languages, including Japanese, Korean, German, Italian, Spanish, Greek, and Hebrew. Although widely used, the eHEALS’ validity has been questioned, mainly due to the lack of correlation between eHEALS scores and actual task performance in online health information seeking [10,11], and because it does not sufficiently address critical and interactive health literacy skills [12,13]. Cameron Norman, the first author of the eHEALS, has also expressed some concern as to whether the scale is able to measure eHealth Literacy in its totality in a world that has witnessed the rise of Web 2.0, and that is generally characterized by the use of ever-changing technology: “The fundamental collection of skills that comprise eHealth Literacy have not likely changed, but the contexts in which they are expressed (...) have” [12] (p. 3). This illustrates the dynamic nature of the concept of eHealth Literacy, and thus also of Media Health Literacy, as both terms qualify skill sets that can only be understood and analyzed within the media environment in which they are applied.

## 3. Media Health Literacy and eHealth Literacy in Context

The media, and especially the realm of digital media, constitute a complex social environment to be navigated by consumers in order to promote and maintain health using the information available in this environment. Tasks related to Media and eHealth Literacy are thus by no means trivial. In order to understand how demanding they are, we illustrate the multi-faceted nature with a case study before focusing on context variables associated with Media Health Literacy and eHealth Literacy task performance.

Chan and Kaufman [14] used Cognitive Task Analysis to map consumers’ performance during information-seeking and decision-making tasks involving eHealth tools. To disentangle knowledge, thought processes and skills necessary for task completion, they coded every reported step in a matrix involving facets of eHealth Literacy and levels of cognitive complexity. They drew on Norman and Skinner’s [4] Lily model which postulates that eHealth Literacy combines six literacy domains: traditional literacy, information literacy, scientific literacy, media literacy, computer literacy, and health literacy. Any given eHealth Literacy task requires a certain degree of skills and knowledge in the said areas. In their study, detailed analyses of performance in a six-step task involving a consumer health webpage showed that any step required skills from at least two literacy domains, often more, with the cognitive complexity most often rated 4 or 5 out of 6 levels by experts. The most frequently identified barriers to task completion were encountered with steps requiring information and computer literacy. Surprisingly, the majority of challenges faced by participants fell within the lower ranges of cognitive demands.

**Figure 1:**
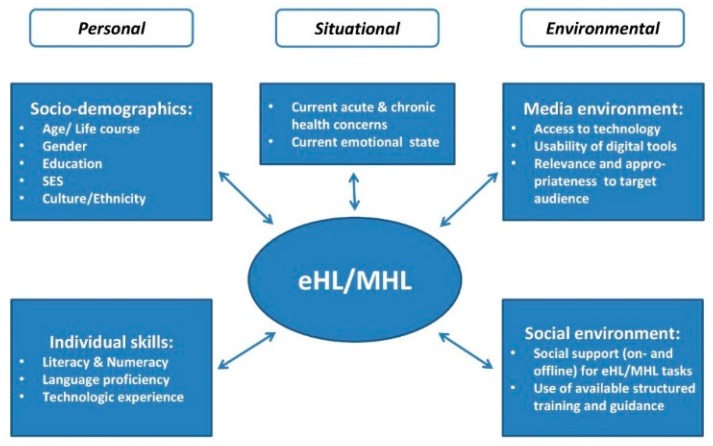
The complexity of eHealth Literacy (eHL) and Media Health Literacy (MHL) in context.

This example demonstrates that eHealth Literacy, as mentioned, is by no means a trivial set of skills in a highly digitalized environment. On the contrary, it combines knowledge and skills from a wide variety of domains and is inherently relevant within the social contexts in which Health Literacy, Media Health Literacy, and eHealth Literacy are developed and applied by an individual or group. The following sections, as illustrated in Figure 1, will elaborate on what is known regarding how factors in an ecological model affect Media Health Literacy and eHealth Literacy. Growing academic attention has been given to system complexity, personal and socio-demographic factors such as age, gender, and education, social environment and context that together play a major role in shaping skills in performing health literacy related tasks in digital media environments.

### 3.1. Complexity of Systems and Environments

In 2009, Parker [15] made an important statement that is occasionally forgotten in a discourse that focuses its attention predominantly on health literacy as an individual combination of skills: “One must align skills and abilities with the demands and complexities of the system” (p. 92). She illustrated this with a simple drawing of two arrows pointing toward each other, one representing “skills/abilities” and the other labelled “demands/complexity”. Where the two arrows meet, she wrote, is where health literacy is expressed.

Digital media sources of health information have particular potential to reduce system complexity. Usability and accessibility are topics that receive specific attention from software developers and web designers. Several findings suggest that focusing on user experience and designing with the aim of reducing complexity are beneficial for digital health literacy. For example, disadvantage in written and spoken language skills can be barriers to accessing online health information [16]. Information should, therefore, be made increasingly available in more interactive formats that depend less on formal literacy and knowledge of the local language [17]. Meppelink and colleagues [18] provide empirical support for this claim. In an experimental study they show that recall and attitude change were significantly higher in low health literate participants when information was presented verbally and enriched with animations supporting the content compared to standard written text and illustrations. Content must be adapted to be relevant to the specific population, for example taking into consideration cultural eating habits when designing a smartphone app to support weight loss [19]. Thus, (digital) media solutions for health actually do have the potential to contribute to making health information more accessible and understandable for broad sections of the population, eventually fostering positive effects on health [20].

System complexity is also reduced when people become more experienced with health literacy tasks and with the technology that can be used to apply health literacy skills. Accordingly, eHealth Literacy scores are positively associated with frequency of use of the Internet [21,22] and with the number of Web searches for health information [23]. High eHealth Literacy levels are associated with the use of social media for the purpose of seeking health information, and with frequent use of electronic devices in general [24]. It can also be shown that eHealth Literacy scores are higher for students who had been actively involved in searching for health information online than for non-experienced peers [25]. Similarly, data suggest that parental online health information seeking is positively associated with adolescents’ eHealth Literacy and engagement in online searches for health information [26]. These findings support the conclusion that eHealth Literacy skills are strongly shaped by exposure to technology, the Internet, and online health information sources in particular. It may therefore be deduced that the higher the usability of the underlying technology, i.e., reducing system complexity, the greater the exposure, and the greater the engagement of digital resources by the population.

### 3.2. The Role of Socio-Demographics

A number of socio-demographic variables are linked to Media Health Literacy, and specifically to online health information seeking and eHealth Literacy, measured at the individual level. Media Health Literacy, to date, measured mainly among adolescents, is highly associated with socioeconomic status (SES) and mothers’ level of education [3]. Regarding digital sources of health information, people from different age groups, socioeconomic backgrounds, and from diverse ethnic groups refer to online sources when looking for information on health topics [27]. As early as 2006, 80 percent of adult American Internet users confirmed to have browsed the Web for health information [28]. Similar numbers of online health information seeking have more recently been shown in Eurobarometer data from 28 member states of the European Union [29]. American college students even seem to consider the Internet as their single most important source of health information [30]. Still, studies also identified some socioeconomic differences in online health information seeking. Low rates of online health information seeking were reported among older adults, among people with low educational attainment, and in men compared to women [31,32,33,34]. Regarding the use of eHealth tools among ethnic minorities, the data is inconclusive. According to recent studies, as opposed to previous ones, no significant differences between groups have been evidenced [35]. Yet, the cultural context of eHealth literacy including mobile health (mHealth) has been recognized [36].

According to Neter and Brainin [37], people with high eHealth Literacy are younger and better educated than people with low eHealth Literacy scores. These associations of eHealth Literacy with age and education are confirmed by data from various samples, e.g., financially disadvantaged US families [38] and immigrant communities in Canada [39]. These socio-demographic differences are consistent for mHealth use, health literacy, eHealth Literacy, and Media Health Literacy, particularly with regard to education and age, and secondarily with regard to gender and ethnic background. Cultural background has also been considered to significantly influence eHealth Literacy and Media Health Literacy such that researchers in South Korea [40] and Italy [41], conducted several validation studies for the eHEALS model to assure its relevancy to local culture.

### 3.3. Social Networks

Socio-demographics and experience with media and technology are factors on the individual level that influence eHealth Literacy and Media Health Literacy skill sets. Certainly, individual level variables contribute to shaping health literacy levels. However, caution is warranted as to “the individualistic premise of current literature (on health literacy) in which individuals are treated as isolated and passive actors” [42] (p. 1309). Several findings suggest that eHealth Literacy levels are shaped and can possibly be improved through guidance in online health information seeking activities by more experienced users as well as in structured learning environments. For example, Chang and colleagues [26] showed that active parental mediation of their adolescent children’s Internet use predicted adolescents’ eHealth Literacy. Participants in focus groups conducted among Spanish primary school students reported use of the Internet as a tool for learning about health topics and habits, but preferred their searches to be guided and supervised by their parents to promote their efficacy and confidence in dealing with online (health) content [43]. Similarly, in a sample of elderly living with chronic disease, participants reported the Internet as a useful information source on their condition. Still, they often relied on the help of relatives and friends when assessing the information [44]. A similar strategy has been observed for Hispanic breast cancer survivors in the United States; managing online health information in their case was a responsibility they consistently shared with their offline social networks [45]. Results from a nationally representative Israeli survey indicate that participants with low eHealth Literacy for whom finding someone (offline) to help them perform and analyze their online health information searches was easy, partly compensated for their lack of proficiency with digital health literacy through social support [46]. Caregivers’ or significant others’ guidance and support are thus vital in the development of abilities relevant to eHealth Literacy in context.

## 4. Improving Media Health Literacy and eHealth Literacy

Studies focused on the implementation and effectiveness of Media Health Literacy and eHealth literacy training programs, are relatively few. Regarding Media Health Literacy, as it inherently includes exercising critical thinking, and acknowledging that new channels of intervention need to be developed and applied for health promotion among adolescents, Wharf Higgins and Begoray [47] developed the concept of Critical Media Health Literacy. The concept focuses on attributes that include skill sets, empowerment, and competency of engaged citizenship. While the conceptual basis has been established, related intervention has been tested primarily on children and adolescents, focusing on media literacy related to health topics, e.g., alcohol [48]. Among adults, health literacy has been incorporated into media driven interventions, to learn of the differential effects of low and high health literacy. In order to influence the consumption of sugar sweetened beverages among the rural community in the US, a media driven intervention was developed and implemented while measuring the effects among various levels of health literacy. The program was found to be just as effective among participants with low health literacy as compared to high health literacy [49]. Media health literacy has also been given serious attention not only by public health entities, but also by media stakeholders, just as journalists, exemplified by the seriousness with which news media serves the public’s health literacy needs while influencing public health policy as well [50]. Still, interventions aimed at improving Media Health Literacy across the lifespan, based on, and including critical health literacy, have yet to take a prominent place in intervention research.

Regarding eHealth literacy, a systematic review on eHealth Literacy among college students concluded that even this young, well-educated population has major shortcomings, the findings of which show that interventions to improve eHealth Literacy would not only benefit traditional at-risk groups [51]. While literature on interventions aiming to improve digital health literacy is scarce to date, some promising findings have been published. eHealth Literacy can be developed and improved by offering structured learning opportunities. For example, an intervention to improve eHealth Literacy among adolescents composed of three online training lessons yielded significant, though marginal improvements of digital health literacy levels among the participants. High identification with, and involvement in the intervention, i.e., feeling that improving eHealth Literacy was important and relevant, was one of the strongest predictors of changes in skill level, stressing the need to make eHealth Literacy personally relevant to potential intervention participants [52]. An intervention consisting of four two-hour sessions aimed at helping older adults perform online health information searches yielded significant improvements of eHealth Literacy from pre- to post-intervention. Participants also reported changes in health-related attitudes and behaviors following the intervention [53,54,55]. It should, however, be noted that a systematic review on eHealth Literacy intervention studies for older adults [56] concluded that many studies apply weak study designs and that some interventions lack a thorough theoretical base. Therefore, further research in the area is greatly needed. Likewise, it should be noted that the reported interventions are primarily skill-based interventions aimed at increasing individual competence. This type of intervention has its justification, however, coupling with interventions focusing more on empowerment and change in the environment where health literacy is applied, is of great importance in an increasingly digitized and media-saturated environment. Finally, as mentioned, reducing system complexity and improving the accessibility of new health technologies and media content ultimately benefit the general population, not only those with low levels of Media or eHealth Literacy.

## 5. Ethical Considerations in Media and eHealth Literacy Practice, Research, and Policy

The need for ethical considerations is just as pertinent and imminent in the areas of media and digital health literacy as in all areas of public health research. Ethical concerns need to be considered comprehensively—in practice, research, and policy.

### 5.1. Media and eHealth Literacy Ethics in Research

Regarding the ethical considerations of research on eHealth and digital/Media Health Literacy, two main aspects need to be considered for ethical scrutiny—namely sampling framework and generalizability of results. Increasingly, public health research relies on both samples that are drawn from big data, and self-reporting through digital systems. In normal research protocol, the use of personal data would require the consent of the participants. The use of big data systems for sampling should comply with the same standards even though the data is usually not identified [57]. Secondly, using digital technology (e.g., Smartwatches, fitness trackers) for data collection can seriously limit the extent to which data is collected from digitally excluded populations, often under-representing those whom have already been mentioned to tend to have low eHealth Literacy and Media Health Literacy. Thus, the results of such research cannot claim to be valid for all populations, nor is the principle of equity in research upheld.

### 5.2. Media and eHealth Literacy Ethics in Practice and Policy

As mentioned above, interventions with regard to MHL and eHealth literacy have two focal aspects: improving these areas of health literacy and/or adjusting interventions so that they are appropriate for the diversity of Media Health Literacy and eHealth Literacy skills. As such, ethical practice needs to be exercised as in any intervention, and applied to Media and eHealth Literacy practice. Intervention in the digital world requires that special attention be given to equity, allowing access according to need, guaranteeing cultural appropriateness, overcoming the digital divide, and taking into consideration various stages of digital development. Whether the intervention is through the digital media or in non-digital media, the characters, storyline, visuals, and content must be population appropriate. Finally, as the media and digital worlds attract commercial investors, public health practitioners must exercise scrupulous ethical standards in order to guarantee that no commercial vested interest is influencing any aspect of the intervention.

In light of all of the above, and in the interests of equity, it is essential that policies for health promotion, for improving health literacy of the individual, and for promoting organizational health literacy for the population, take into account the diversity of Media and eHealth Literacy skill levels.

## 6. Discussion

Media Health Literacy and eHealth Literacy are two concepts closely linked to health literacy which is defined as skills and competencies that enable people to obtain and interpret health information and empower them to maintain and improve their health and the health of the people around them. In Media and eHealth Literacy, the sources of the said health information and tools are specified to be the media, or in the case of eHealth Literacy more specifically digital media. Identifying, extracting, and understanding health information from media sources are by no means straightforward tasks, even less when the information is to be applied, leading to health decisions and adoption or change of health behavior. The complexity of processes underlying health literacy tasks explains why contextual and environmental variables play such an important role in shaping both the development and the actual use of the necessary skill sets.

Several research findings have indicated that health literacy levels vary by educational background e.g., [58,59], and similar findings have been summarized for eHealth Literacy and Media Health Literacy e.g., [3], in earlier sections of this article. This may be the result of education acting as an SES proxy [35], as well as skill sets developed through educational settings in the lifespan. The latter is a standpoint supported by scholars who closely link the development of health literacy to school health education [6,60]. Still, caution needs to be exercised neither to interpret these findings as limitations of populations with low educational backgrounds, nor to conclude that formal education is the only key to improving general health literacy, Media Health Literacy and eHealth Literacy.

Beyond education, studies on general eHealth Literacy have repeatedly shown that the more often an individual engages in the search and interpretation of health information, the more confident they feel doing so. This has yet to be specifically measured for Media Health Literacy. A more overarching conclusion would thus be that self-efficacy [61], a strong predictor of health behavior adoption, is relevant for the eHL and MHL skills sets as well, supported by experience in the lifespan (“practice makes perfect”). It thus may be of secondary importance whether this practice is acquired in structured learning environments provided by formal education or elsewhere. As a third conclusion from findings summarized previously, it can be understood that social support is paramount for many, in executing tasks related to health information from media sources. Over a decade ago, Lee, Arozullah, and Cho [42] proposed a research agenda that would examine the associations of health literacy, social support, and health outcomes. Several studies have researched this assumption, with interesting results. For example, de Wit and colleagues [62] conducted a meta-analysis showing that social support and co-learning in communities were essential for critical health literacy based on qualitative evidence.

Furthermore, not only the social relevance of the practice of health literacy related tasks is of great importance, but also system complexity. Digital and non-digital media—and any other—environments where people encounter health-related information, vary greatly as to how difficult they are to interpret and navigate. Options exist to reduce complexity of content and presentation mode, as some examples introduced above can corroborate. It is the joint responsibility of public health researchers and practitioners, policy makers, and developers to apply what is known and to monitor whether necessary changes in system complexity are applied, leading to ease of access and usability for the actual end users. Thus, not only technical accessibility but also the content and modes of presentation of health information in the media are crucial. Specialists in health promotion, health technology, and health communication need to work together to create the tools that will empower patients to take responsibility for their health [63].

While an abundance of studies has been published in recent years on eHealth Literacy and Media Health Literacy, several limitations are noted, namely the lack of real-time surveys of usage, the response rates not reflecting the majority of users (30–35% response rates) and lack of research studying causal pathways (currently most studies are cross-sectional). In addition, comparative studies between Media and eHealth Literacy may be limited, as general Media Health Literacy includes media that are often not interactive, such as television, while specifically digital media is predominantly interactive.

Lastly, the media are unfortunately subjugated to vast commercial interests that in many cases conflict with the best health interest of consumers. As mentioned above, this leads to very pertinent ethical challenges in the realm of Media and eHealth Literacy research, further stressing the need for inter-sectorial cooperation and involvement of political stakeholders in the discourse on health literacy in media environments.

## 7. Conclusions

The influence of the social environment on public health is significant, as shown in a wealth of studies. As society and the social environment on the global level increasingly move towards use of digital and media tools for delivering health messages, offering health information, navigating health services, while also increasing the use of the Internet for commercial advertising, then eHealth literacy and Media Health Literacy skills will likewise play an increasingly essential role. eHealth Literacy has taken Media Health Literacy to a different level of meaning, as it enables and invites the public to actively interact, respond, and participate in creating, criticizing, and sharing health messages and information. Future research needs to be expanded to understand the symbiotic relationship between Media Health Literacy, eHealth Literacy, and the social and cultural environment. On the one hand, a clearer understanding is necessary to learn of how Media and eHealth Literacy can influence the social environment that promotes health, while also taking into consideration the influence of the social and cultural environment on all aspects of the involved skill sets. The pervasive and increasing access to mobile tools globally will ultimately transform what was once considered the “digital divide” into numerous degrees of “digital development”. Continued concern must be exercised to enable and ensure access to media and digital tools for all, such that new technologies can fulfil their primary purpose: to promote health.
